# Outcomes of Pancreas‐Sparing Total Duodenectomy for Severe Duodenal Polyposis in Patients With Familial Adenomatous Polyposis

**DOI:** 10.1002/ags3.70125

**Published:** 2025-11-21

**Authors:** Takehiro Shiraishi, Hideyuki Ishida, Takatoshi Matsuyama, Noriyasu Chika, Yoshiko Mori, Norimichi Chiyonobu, Youichi Kumagai, Ibuki Fujinuma, Toshiro Ogura

**Affiliations:** ^1^ Department of Digestive Tract and General Surgery, Saitama Medical Center Saitama Medical University Saitama Japan; ^2^ Department of Clinical Genetics, Saitama Medical Center Saitama Medical University Saitama Japan; ^3^ Department of Gastroenterological Surgery Saitama Cancer Center Saitama Japan

**Keywords:** duodenal polyposis, familial adenomatous polyposis, pancreas‐sparing total duodenectomy

## Abstract

**Aim:**

Spigelman stage IV duodenal polyposis (SP‐stage IV DP) is associated with high duodenal cancer risk in patients with familial adenomatous polyposis (FAP). This study evaluated the surgical and oncological outcomes of pancreas‐sparing total duodenectomy (PSTD) as a surgical prophylaxis for severe duodenal polyposis in FAP.

**Methods:**

Medical records were reviewed to evaluate factors concerning short‐ and long‐term clinical and oncological outcomes in consecutive patients with FAP who underwent PSTD for SP‐stage IV DP.

**Results:**

There were twenty‐seven patients (median age: 48 years) from 26 families, of whom 12 were female. Clavien–Dindo grade IIIa/IIIb complications included delayed gastric emptying (*n* = 14) and pancreatic fistula (*n* = 10); no mortalities were observed. Histopathological examinations revealed no malignant neoplasms deeper than T1a in the duodenum and ampulla. Follow‐up (median 6.4 years) revealed anastomotic stricture of the reconstructed neo‐common channel (*n* = 5), anastomotic ulcer of the gastrojejunostomy site (*n* = 5), acute pancreatitis (*n* = 4), and acute cholangitis (*n* = 2), all of which were successfully treated endoscopically or conservatively. Malignant neoplasms after PSTD included gastric cancer (*n* = 3), remnant ano‐rectal cancer (*n* = 3), ileal cancer (*n* = 1), ileal pouch cancer (*n* = 1), and endometrial cancer (*n* = 1). The cumulative 10‐year survival rate following PSTD was 87.4%.

**Conclusions:**

PSTD for the prophylactic management of SP‐stage IV DP was associated with notable but manageable postoperative morbidity. Long‐term surveillance remains essential for the development of extraduodenal malignancies to confirm the oncological efficacy of this type of surgery.

## Abstract 2

This study evaluated the surgical and oncological outcomes of pancreas‐sparing total duodenectomy (PSTD) as a surgical prophylaxis for severe duodenal polyposis in 27 cases of FAP.

## 1 Introduction

Familial adenomatous polyposis (FAP) is an autosomal dominant disorder typically characterized by hundreds to thousands of colorectal adenomas. Prophylactic (procto)colectomy is the gold standard therapy among these patients, because colorectal cancer develops in more than 90% of patients if left untreated [1].

Duodenal cancer (DC) is the third leading cause of death in patients with FAP [2]. Patients with FAP have a 39.2% and 7.7% lifetime risk (up to 50 years old) of developing duodenal polyposis (DP) and DC, respectively [3]. FAP‐associated DP is considered a precursor lesion of DC. In 1989, Spigelman et al. [4] developed a classification system for evaluating FAP‐associated DP. However, a modification introduced in 2004, based on a revised classification for adenomas [5, 6], is now widely used. Spigelman stage IV (SP‐stage IV) DP is considered a severe type of DP that is associated with a high risk of DC [7]. For these patients, the clinical guidelines [8] recommend close endoscopic surveillance by experts or prophylactic surgery such as pancreatoduodenectomy (PD), pylorus‐preserving PD (PPPD), or pancreas‐sparing duodenectomy (PSD). Surgical procedures involving complete duodenal resection without preservation of the pyloric ring are called pancreas‐sparing total duodenectomy (PSTD) or pancreas‐preserving total duodenectomy.

Among these three surgical approaches, PSD is ideal because it avoids the oncologically unnecessary resection of the pancreatic head. Thus, PSD has been preferentially performed in patents with FAP [9], but the extent of duodenal resection and the types of reconstruction associated with PSD widely vary. At our institution, we have performed PSD for SP‐stage IV DP in patients with FAP since 2013 [10]. Our policy for PSD includes total removal of the duodenum (i.e., PSTD) to completely prevent DC and straightforward reconstruction with Billroth type I gastrojejunostomy to enable an easier endoscopic approach for the stricture of bilio‐jejunal and/or pancreato‐jejunal anastomosis, along with long‐term follow‐up of the “neoduodenum.” Although PSD/PSTD for FAP is performed at some specialized facilities, very few reports describe its postoperative complications and short‐ and long‐term oncological outcomes. In this context, this study aimed to clarify the feasibility, safety, and oncological outcomes of PSTD for SP‐stage IV DP in patients with FAP.

## 2 Patients and Methods

### 2.1 Design and Study Population

This single‐center retrospective cohort study included consecutive patients with FAP who underwent PSTD for preoperatively diagnosed SP‐stage IV DP between June 2013 and November 2024 at the Department of Digestive Tract and General Surgery, Saitama Medical School, Saitama Medical University. All the patients were confirmed to have germline pathogenic variants in the *APC*. This study was approved by the local ethics committee of Saitama Medical School, Saitama Medical University (No. 925‐IX, No. 1355‐VIII). Informed consent was obtained from all patients.

### 2.2 Data Collection

From the medical records, we retrospectively collected patient characteristics, pre‐ and postoperative pathological findings, postoperative short‐ and long‐term complications, occurrence of postoperative neoplastic lesions, and survival time. The Clavien–Dindo (CD) classification was used to evaluate postoperative complications. Postoperative pancreatic fistula and delayed gastric emptying (DGE) were defined by the International Study Group for Pancreatic Fistula (ISGPF) grading [11] and International Study Group for Pancreatic Surgery [12], respectively. Complications were considered short‐term if they occurred within 30 days of operation and long‐term for those that occurred thereafter. Most patients were followed up at our institution, and long‐term outcomes were documented. At our institution, patients were seen 2–3 times a year for at least 5 years after surgery, during which endoscopic examinations for FAP were also performed as surveillance [8]. For patients who received postoperative care at other hospitals, the postoperative complications and oncological information were obtained from the treating physicians as appropriate. Of these, the initial 10 consecutive cases were previously reported with a focus on surgical feasibility and postoperative morbidity [10]. Additionally, 24 cases—including the initial 10—were presented in a separate study comparing PSTD and PD concerning postoperative nutritional outcomes [13].

### 2.3 Preoperative Endoscopic Assessment

High‐definition endoscopes with advanced imaging techniques (e.g., narrow band imaging) were used to assess the size and mucosal pattern of the adenomas. An endocytoscopy system was occasionally used to observe and evaluate duodenal lesions [14]. The Spigelman classification was used as previously described [4]; the number, size (maximal diameter), histology (tubular, tubullo‐villous, or villous), and severity of dysplasia (low or high grade) of adenomas are assessed from 1 to 3, with the total score determining the disease stage. Gastroduodenoscopy and pathological examination were carried out by board‐certified specialists. The Spigelman classification was determined by specialists in FAP, polyposis syndromes, gastrointestinal diseases, and hepatopancreatobiliary disorders. PSTD was selected as the preferred surgical approach for SP‐stage IV neoplasms. When ampullary lesions with high‐grade dysplasia potentially invade beyond the mucosa (deeper than T1) [15], preoperative endoscopic ultrasonography was routinely performed to assess tumor depth and exclude intraductal extension.

### 2.4 Postoperative Surveillance

For 5 years after PSTD, computed tomography was performed at least once a year, upper gastrointestinal endoscopy was performed once a year, and colonoscopy was performed once every 1–2 years. Magnetic resonance cholangiopancreatography was additionally performed when detailed evaluation of the postoperative pancreatobiliary tract was required.

### 2.5 Surgical Technique

Our surgical technique for PSTD was based on that previously described by Watanabe et al. [10], and procedures of PSTD are shown in Figure S1. This begins with an upper midline incision, and cholecystectomy is performed routinely. After accurately locating the ampulla of Vater through cholecystectomy and inserting a catheter from the cystic duct, the duodenum is carefully dissected from the pancreas to expose the proximal ends of the common bile duct and Santorini duct. Afterward, the stomach is transected between the antrum and body, then the proximal jejunum is transected 10–15 cm distal to the Treitz ligament. The extent of gastrectomy varied and included subtotal stomach‐preserving gastrectomy, subtotal gastrectomy, or distal gastrectomy. The surgical approach was selected based on the presence, absence, and anatomical location of adenomas or high‐grade dysplasia within the antrum. After cutting the Wirsung and Santorini ducts adjacent to the duodenal wall, dye is injected into the Wirsung duct to evaluate its communication with the Santorini duct. After the dye‐injection test, the Santorini duct is ligated. To confirm the absence of neoplastic lesions, the common duct stump is submitted for intraoperative frozen section diagnosis. During reconstruction, the proximal jejunum is repositioned in the duodenal bed, and end‐to‐end gastrojejunostomy was performed. After performing septoplasty on the common bile duct and Wirsung duct, an end‐to‐side pancreatojejunostomy is meticulously performed. Blumgart pancreatojejunostomy was performed in 7 out of 11 cases in the last 5 years. Finally, a catheter is inserted into the main pancreatic duct and the bile duct, respectively.

### 2.6 Statistical Analysis

Descriptive statistics were used for demographics, perioperative outcomes, and long‐term outcomes. Categorical variables are presented as numbers and percentages (*n*/%). Continuous variables with normal and skewed distributions, respectively, are presented as means ± standard deviations and medians with ranges. Logistic regression analysis regarding most frequent postoperative complication was subsequently performed to calculate the odds ratio and 95% confidence interval. Overall survival time was defined as the interval from surgery to the last follow‐up or until death. The Kaplan–Meier method was used to calculate survival probabilities. All statistical procedures were performed using the JMP Pro 16.2.0 software (SAS Institute, Cary, NC, USA). All *p*‐values were two‐sided, and *p* < 0.05 was considered statistically significant.

## 3 Results

### 3.1 Study Population

A total of 29 consecutive patients with FAP underwent PSTD during the study period. Of these, one patient required total gastrectomy due to multiple high‐grade dysplastic lesions throughout the stomach. Another patient, with an unclear Spigelman stage diagnosis, underwent intensive downscaling polypectomy at a different hospital. Consequently, 27 patients were eligible for inclusion in the study.

The patients' baseline characteristics are shown in Table 1. There were 15 males and 12 females, with a median age at PSTD of 48 (27–68) years, and no patients had a history of high surgical risk. Prophylactic (procto) colectomy was done in 25/27 patients, with a median age at (procto) colectomy of 33 (17–49) years old. The remaining two patients did not undergo any surgical intervention and instead underwent repeated intensive downstaging polypectomy [16] and careful surveillance by an endoscopic specialist. A history of malignant neoplasms was present in eight patients; however, all of these cancers had been completely resected before the PSTD.

**TABLE 1:** Baseline characteristics.

Parameters	Categories	Total (*n* = 27)
Age (years) at PSTD, median (range)		48 (27–68)
Sex, *n* (%)	Male	15 (56)
	Female	12 (44)
Body mass index, median (range)		22.5 (15.6–28.1)
History of (procto) colectomy, *n* (%)	TPC	1 (4)
	IRA	10 (37)
	IPAA	14 (52)
	None^a^	2 (7)
Age at (procto)colectomy, median (range)		33 (17–49)
History of intra‐abdominal desmoid tumor, *n* (%)		5 (19)
History of malignant neoplasms, *n* (%)	Colorectal cancer	3 (11)
	Gastric cancer	1 (4)
	Thyroid cancer	2 (7)
	Seminoma	1 (4)
	Ovarian cancer^b^	1 (4)
	Endometrial cancer^b^	1 (4)

*Note:* TPC total proctocolectomy with permanent ileostomy. IRA total colectomy with an ileorectal anastomosis. IPAA restorative proctocolectomy with an ileal pouch‐anal anastomosis.

^a^ Repeated intensive downstaging polypectomy and careful surveillance by an endoscopic specialist.

^b^ One patient experienced both ovarian and endometrial cancers.

### 3.2 Perioperative Outcomes and Postoperative Complications

All patients underwent PSTD via an open surgical approach. The perioperative outcomes are summarized in Table 2. The median operative time was 378 min (range: 281–710 min), and the median estimated blood loss was 443 mL (100–2000 mL). The median postoperative hospital stay was 35 days (11–98 days). Histopathological findings are also detailed in Table 2.

**TABLE 2:** Peri‐operative outcomes and pathological outcomes.

Parameters	Total (*n* = 27)
Postoperative hospital stay, day (range)	35 (11–98)
Duration of surgery, minutes (range)	378 (281–710)
Blood loss, mL (range)	443 (100–2000)
Perioperative compliction, *n* (%)	
Type of complication	
Pancreatic fistula grade A^a^	3 (11)
Pancreatic fistula grade B^a^	10 (37)
Delayed gastric emptying	14 (52)
Leakage of gastrojejunostomy	2 (7)
Acute pancreatitis	4 (15)
Acute cholangitis	7 (26)
Wound infection	5 (19)
No complication	3 (11)
Clavien‐Dindo classification	
Grade I‐II	10 (37)
Grade IIIa	13 (48)
Grade IIIb	1 (4)
Reoperation, *n* (%)	1 (4)
Histopathology, *n* (%)	
Duodenum	
Adenoma	18 (67)
Adenocarcinoma	Tis:1 (4) T1a:6 (22)
Duodenal papilla	
Adenoma	7 (26)
Adenocarcinoma	T1a:1 (4)
NET G1^b^	1 (4)

^a^ The International Study Group for Pancreatic Fistula (ISGPF) grading.

^b^ WHO Classification of Tumors of the Digestive System 4th Edition 2010.

CD grade IIIa/IIIb complications were seen in 52%. DGE (*n* = 14) and ISGPF grade B^11^ pancreatic fistulas (*n* = 10) were relatively common, all of which resolved with conservative management. The other perioperative complications included acute cholangitis (*n* = 7), acute pancreatitis (*n* = 4), and wound infection (*n* = 5). Only one patient required reoperation related to postoperative bleeding from the omental artery.

We secondarily investigated factors that cause DGE which was the most frequent postoperative complication. The following covariant factors were analyzed: patient age, sex, body mass index, operative time, blood loss, and the presence or absence of postoperative pancreatic leakage. Univariate logistic regression analysis revealed no statistical factors (Table 3).

**TABLE 3:** Univariate logistic regression analysis of risk factors for delayed gastric emptying.

Variables	Odds ratio	95% confidence interval	*p*
Age (years)	0.62	0.03–12.0	0.75
Sex (female)	2.1	0.45–10.4	0.34
Body mass index (kg/m^2^)	0.25	0.007–6.2	0.39
Duration of surgery (minutes)	2.8	0.09–169.3	0.56
Blood loss (mL)	16.9	0.24–7063.7	0.06
Pancreatic fistula (grade B)^a^	0.59	0.11–2.85	0.51

^a^ The International Study Group for Pancreatic Fistula (ISGPF) grading.

### 3.3 Long‐Term Complications and Mortality

Table 4 shows the long‐term complications. The median time interval from surgery was 6.4 (0.5–10.9) years. The long‐term complications included anastomotic stricture of the reconstructed neo‐common channel (*n* = 5, 19%), anastomotic ulcer at the site of gastrojejunostomy (*n* = 5, 19%), acute pancreatitis (*n* = 4, 15%), and acute cholangitis (*n* = 2, 7%). All patients with anastomotic stricture of the reconstructed neo‐common channel were successfully treated with endoscopic balloon dilation and/or stenting. Endoscopic treatments were performed without any problems in all patients. No patients required surgery for pancreatitis or cholangitis, and none developed diabetes mellitus.

**TABLE 4:** Long‐term complications.

Parameters	Total (*n* = 27)
Acute pancreatitis, *n* (%)	4 (15)
Acute cholangitis, *n* (%)	2 (7)
Diabetes mellitus, *n* (%)	0 (0)
Anastomotic ulcer at the site of gastrojejunostomy, *n* (%)	5 (19)
Anastomotic stricture of the reconstructed neo‐common channel, *n* (%)	5 (19)
No complication, *n* (%)	16 (59)

With a median follow‐up period after PSTD of 6.4 years, 8 patients developed malignant neoplasms, including gastric cancer (*n* = 3), remnant rectal cancer (*n* = 3, with 1 case of cancer at the anal transition zone), ileal cancer (*n* = 1), ileal pouch cancer (*n* = 1), and endometrial cancer (*n* = 1). No recurrence of duodenal or ampullary cancer was observed.

**FIGURE 1:**
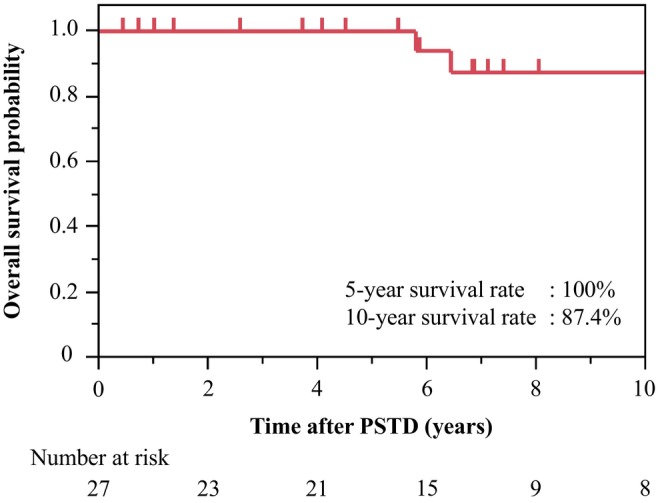
Kaplan–Meier curve indicating the cumulative overall survival after pancreas‐sparing total duodenectomy (PSTD). In this cohort, 3 out of 27 patients died. The cumulative 10‐year survival rate after PSTD was 87.4%.

Table 5 shows the long‐term mortality. A total of 3 patients (11%) died at a median of 6.4 (5.8–10.1) years after PSTD. One patient developed gastric cancer (pathological stage IIIB^15^) and underwent total gastrectomy 5.3 years after PSTD. Subsequently, remnant rectal cancer was detected 6.2 years after PSTD during postoperative adjuvant chemotherapy. Finally, the patient deteriorated and died 6.4 years after PSTD (67 years old). Another patient developed cancer at the anal transition zone with multiple synchronous pulmonary metastases and died 10.1 years after PSTD (56 years old). The last patient was diagnosed with ileal pouch cancer, showing multiple synchronous liver metastases. He died of the disease 5.8 years after PSTD (59 years old). The cumulative 10‐year survival rate after PSTD in all patients (*n* = 27) was 87.4% (Figure 1). None of the mortalities were directly associated with the PSTD.

**TABLE 5:** Malignant neoplasms that developed after PSTD.

Case number	Age (years) at PSTD	Sex	Years after PSTD to tumor detection	Types of cancer
1	51	Male	4.0	Ileal pouch cancer, multiple liver metastases^b^
2	50	Female	8.5	Cancer at the anal transition zone, multiple pulmonary metastases^b^
3	52	Female	4.7	Gastric cancer (T1a x 2 lesions)^a^
			7.3	Endometrial cancer (T1aN0M0, StagIA)^a^
4	59	Female	6.4	Cancer at the ileostomy (T3N0M0, Stage IIA)^a^
5	68	Male	8.4	Remnant rectal cancer (Tis)^a^
6	30	Male	7.5	Remnant rectal cancer (Tis)^a^
7	60	Female	5.3	Gastric cancer (T3N3aM0, Stage IIIB)^a^, ^b^
			6.2	Remnant rectal cancer
8	46	Female	1.6	Gastric cancer (T1aN0M0, Stage IA)^a^

^a^ TNM classification of malignant tumors, 8th edition.

^b^ Mortality.

## 4 Discussion

PSTD was associated with significant yet manageable early postoperative morbidity, including DGE and pancreatic fistula. Additionally, long‐term complications—such as anastomotic stricture of the reconstructed neo‐common channel, acute pancreatitis, and gastrojejunal anastomotic ulcer—were effectively managed through endoscopic interventions or resolved with conservative treatment. The estimated cumulative 10‐year survival rate of 87.4% following PSTD was also confirmed, with no recurrence of duodenal or ampullary cancer. However, various neoplasms developed postoperatively. Therefore, PSTD is oncologically beneficial as a prophylactic surgical approach for advanced duodenal cancer in patients with FAP and severe DP. The notable yet manageable early and long‐term morbidities may be justified by the long‐term oncological advantages of the procedure.

In 1995, Chung et al. [17] first described the indications, surgical techniques, and results of PSD in 5 patients. Four patients with FAP were included in their study, with one having duodenal trauma. The Cleveland Clinic has reported 45 cases of FAP over the past 28 years, among which 27 cases of SP‐stage IV DP were treated with PSD [18], which is the largest number of cases of SP‐stage IV DP in the literature. Similarly, our report included 27 patients with SP‐stage IV DP, but PSTD was the chosen procedure. PSTD was selected over PSD due to the fundamental idea of risk‐reducing surgery, to completely control the cancer risk of the duodenum. Yoon et al. [19] reported that preserving the pylorus and bulbus can be disadvantageous due to the remaining polyps or the future development of polyps in the bulbus, which are difficult to remove endoscopically. Moreover, one case report [20] has described a patient with FAP who developed cancer in the bulbus after pylorus‐preserving pancreatoduodenectomy. Meanwhile, our PSTD technique employs Billroth type I gastrojejunostomy as the reconstruction method because it can facilitate an endoscopic approach to the anastomotic stenosis between the neo‐common channel and jejunum.

We reviewed previous studies that reported at least five cases of PSD or PSTD in patients with FAP, including data on complications and survival outcomes (Table 6) [21, 22, 23, 24, 25, 26, 27, 28, 29]. Eight studies encompassing 160 patients were identified, of whom 142 had FAP. Among the procedures performed, 43 patients (28%) underwent total duodenectomy, whereas 111 (72%) underwent subtotal duodenectomy, with transection at the duodenal bulb. Reconstruction was achieved using Billroth types I and II in 80 and 74 cases, respectively. Regarding short‐term complications, acute pancreatitis emerged as a notable postoperative morbidity, occurring in 15% of cases in our cohort and 3.0%–11.1% of cases reported in the reviewed literature. This was presumably related to the intermittent backwash of bile into the pancreatic duct due to the proximity and loss of the sphincter. Nevertheless, the cases of acute pancreatitis in the present study were mild or moderate, and no patient developed persistent organ failure or required necrosectomy. Meanwhile, other reports have shown that DGE occurs in 3.7%–30% of cases, but this occurred in 52% in our study, though the definition of DGE may be different among studies. The incidence of DGE is generally reported to be approximately 24% in PD and 29% in PPPD [30], and various factors are thought to be involved in its causes. We performed a univariate logistic regression analysis of the causes of DGE, but it was difficult to identify significant factors. Various studies have already speculated on its pathogenesis, including a lack of the gastrointestinal hormone motilin, miscoordination between gastric and jejunal motility, and the effects of complications such as pancreatic leakage [31]. Since the entire duodenum is removed in PSTD, it is highly likely that a lack of motilin, which is mainly secreted from the duodenum and promotes gastrointestinal peristalsis, contributes to DGE. Nevertheless, these complications could be managed with minimally invasive or conservative methods. Additionally, the relatively high propensity for DGE after PSTD highlights the need for postoperative enteral support, which we favor by feeding via the gastrojejunostomy tube.

**TABLE 6:** Reports of PSD or PSTD for Spigelman‐stage IV duodenal polyposis with FAP at least 5cases, including present study.

Author	Reported year	Country	Number of cases (with FAP)	Transection of duodenum	Recontruction	90‐day mortality (%)	Morbidity (%)	Long‐term morbidity (%)	Perioperative complications					Long‐term complications		5‐years OS	10‐years OS	Follow‐up months, (range)
									Leakage of gastrojejunostomy	DGE	SSI	Pancreatic fistula	Pancreatitis	Anastomotic ulcer at the site of gastrojejunostomy	Pancreatitis			
Sarmiento et al. [21]	2002	USA	8 (5)	Bulbus	Billroth‐I	0 (0%)	5 (62.5%)	—	—	—	1 (12.5%)	3 (38%)	—	2 (25%)	2 (25%)	100%	—	23 (6–44)
de Vos tot Neverdeen et al. [22]	2003	Holland	6 (6)	—	—	0 (0%)	4 (66.7%)	—	—	—	—	—	—	—	—	—	—	11 (2–15)
Al‐Sarireh et al. [23]	2008	England	12 (6)	Bulbus	Billroth‐I	0 (0%)	6 (50%)	3 (25%)	—	1 (8.3%)	1 (8.3%)	1 (8.3%)	1 (8.3%)	—	1 (8.3%)	—	—	20 (3–75)
Penninga and Svendsen [24]	2011	Denmark	13 (10)	Antrum or Bulbus^a^	Billroth‐I	0 (0%)	6 (46%)	0 (0%)	1 (8%)	—	—	3 (23%)	1 (8%)	—	—	100%	—	56 (2–134)
Rangelova et al. [25]	2015	Sweden	20 (13)	Bulbus	Billroth‐I	0 (0%)	11 (55%)	—	—	2 (10%)	—	3 (15%)	—	—	—	—	—	—
Petra Ganschow et al. [26]	2018	Germany	27 (27)	Bulbus^b^	Billroth‐I^b^	1 (3.7)	15 (55.5%)	—	—	1 (3.7%)	1 (3.7%)	9 (33.3%)	3 (11.1%)	—	6 (22%)	87.1%	74.7%	70 (3–163)
Walsh et al. [27]	2019	USA	44 (44)	Bulbus	Child	0 (0%)	38 (86%)	—	—	13 (30%)	10 (23%)	6 (14%)	3 (7%)	—	7 (16%)	—	—	107
Aelvoet et al. [28]	2022	Netherlands	30 (30)	Antrum	Billroth‐II	0 (0%)	22 (73%)	11 (37%)	4 (13%)	5 (17%)	—	12 (40%)	1 (3%)	6 (20%)	5 (17%)	95.6%^c^	93.3%^c^	125 (61–167)^c^
Present study	2025	Japan	27 (27)	Antrum	Billroth‐I	0 (0%)	24 (89%)	11 (41%)	2 (7%)	14 (52%)	5 (19%)	13 (48%)	4 (15%)	5 (19%)	4 (15%)	100%	87.4%	77 (6–130)

^a^ Resection of duodenal mucosa.

^b^ Although there are no detailed descriptions, it is assumed to be the same surgical procedure as reported by Müller et al. [29] (Report from the same facility).

^c^ Long‐term prognosis has been evaluated in 27 of the 30 cases.

In the Japanese clinical guidelines for the treatment of hereditary colorectal cancer [8], PD, PPPD, and PSD/PSTD are presented with equal merit as surgical resections for severe duodenal polyposis associated with FAP. For surgeons specializing in the hepato‐biliary‐pancreatic region, PD or PPPD is a simpler surgical procedure and may be considered the standard. However, for preventive resection of hereditary tumors, it is considered better to avoid organ resections unrelated to cancer. In fact, a systematic review of PSTD [32] found that of 211 cases in which PSTD was performed, 75% had duodenal polyposis associated with FAP. Although published after we began using PSTD, a retrospective comparison of PD and PSD cases for duodenal adenomas revealed that PSD shortened surgical time compared with PD and did not impair pancreatic exocrine function. Furthermore, no differences were observed between PD and PSD in terms of intraoperative blood loss, postoperative pancreatic fistula, DGE, or surgical site infection. Another study compared nutritional indicators between PSTD for duodenal severe polyposis associated with FAP and PD for low‐malignancy duodenal neoplasms [13], finding that PSD provided better postoperative nutritional status and maintained glucose tolerance than PD.

Regarding long‐term complications, notable morbidities observed after PSTD included anastomotic stricture of the reconstructed neo‐common channel (19%), associated acute pancreatitis (15%), and anastomotic ulcer at the gastrojejunostomy site (19%). All cases were successfully managed with endoscopic treatment. A key advantage of PSTD lies in its anatomical reconstruction of the digestive tract, facilitating endoscopic surveillance compared to the nonanatomical reconstruction associated with the Whipple procedure [33].

While assessing long‐term oncological outcomes, considering FAP‐associated malignancies such as gastric, jejunal, ileal, and anorectal cancers, is crucial. Recent long‐term studies following restorative (procto)colectomy with ileal pouch–anal anastomosis (IPAA) and total colectomy with ileorectal anastomosis (IRA) have reported persistent risks of malignancy in the anal transitional zone or remaining rectum [34, 35]. In our series, four patients developed anorectal cancer following PSTD. Other post‐PSTD malignancies included three gastric cancers, one ileal cancer, one ileal pouch cancer, and one endometrial cancer. Although the relationship between DP severity and subsequent cancer development remains unclear, this warrants further investigation.

Nevertheless, previous reports by Ganschow et al. [26] and Aelvoet et al. [28] on PSD and PSTD for FAP with varying Spigelman stages demonstrated 5‐ and 10‐year survival rates of 87.1%–95.6% and 74.7%–93.3%, respectively, with no duodenal cancer‐related deaths. Each study included 27 patients. In our series, the 10‐year survival rate was 87.4%, reinforcing the notion that PSTD provides long‐term outcomes in patients with FAP and SP‐stage IV DP.

This study has several limitations including an infrequent disease, the single‐center study, and the retrospective design; hence, the outcomes may make information bias and may limit the generalizability of our findings. However, all patients underwent surgery within 1 year of being diagnosed with SP‐stage IV DP, so there was no selection bias. However, considering the rarity of FAP—estimated to occur in 1 in 10 000 to 20 000 individuals—our department's extensive experience with FAP makes this case series a valuable contribution to clinical decision‐making and patient counseling.

## 5 Conclusion

PSTD with Billroth type I gastrojejunostomy for FAP‐associated SP‐stage IV DP is associated with a relatively high rate of postoperative morbidity. However, these complications were successfully managed through endoscopic or conservative treatment. No recurrence of duodenal or ampullary cancer was observed, but long‐term surveillance remains essential for the development of extraduodenal malignancies to confirm the oncological efficacy of this type of surgery.

## Author Contributions

**Takehiro Shiraishi:** conceptualization, methodology, data curation, investigation, writing – original draft, validation, formal analysis, visualization. **Hideyuki Ishida:** data curation, conceptualization, methodology, investigation, validation, supervision, writing – review and editing. **Takatoshi Matsuyama:** investigation, validation, writing – review and editing. **Noriyasu Chika:** investigation, validation, writing – review and editing. **Yoshiko Mori:** investigation, validation, writing – review and editing. **Norimichi Chiyonobu:** investigation, validation, writing – review and editing. **Youichi Kumagai:** investigation, validation, writing – review and editing. **Ibuki Fujinuma:** investigation, validation, writing – review and editing. **Toshiro Ogura:** investigation, validation, writing – review and editing.

## Disclosure

Human and animal rights: This article does not contain any studies with animals performed by any of the authors.

## Ethics Statement

All procedures performed in studies involving human participants were in accordance with the ethical standards of the institutional and/or national research committee and with the 1964 Helsinki declaration and its later amendments or comparable ethical standards.

## Consent

Informed consent was obtained from all the individual participants included in the study.

## Conflicts of Interest

The authors declare no conflicts of interest.

## Supporting information


**Figure S1:** Procedures of pancreas‐sparing total duodenectomy.
