# Supplementary material for: Outcomes of Pancreas‐Sparing Total Duodenectomy for Severe Duodenal Polyposis in Patients With Familial Adenomatous Polyposis
Source: Ann Gastroenterol Surg. 2025 Nov 21;10(2):534–42. doi: 10.1002/ags3.70125 (PMC12962038; doi:10.1002/ags3.70125)
Supplement: Supplementary file 1 — Figure S1: Procedures of pancreas‐sparing total duodenectomy. [file AGS3-10-534-s001.pdf]

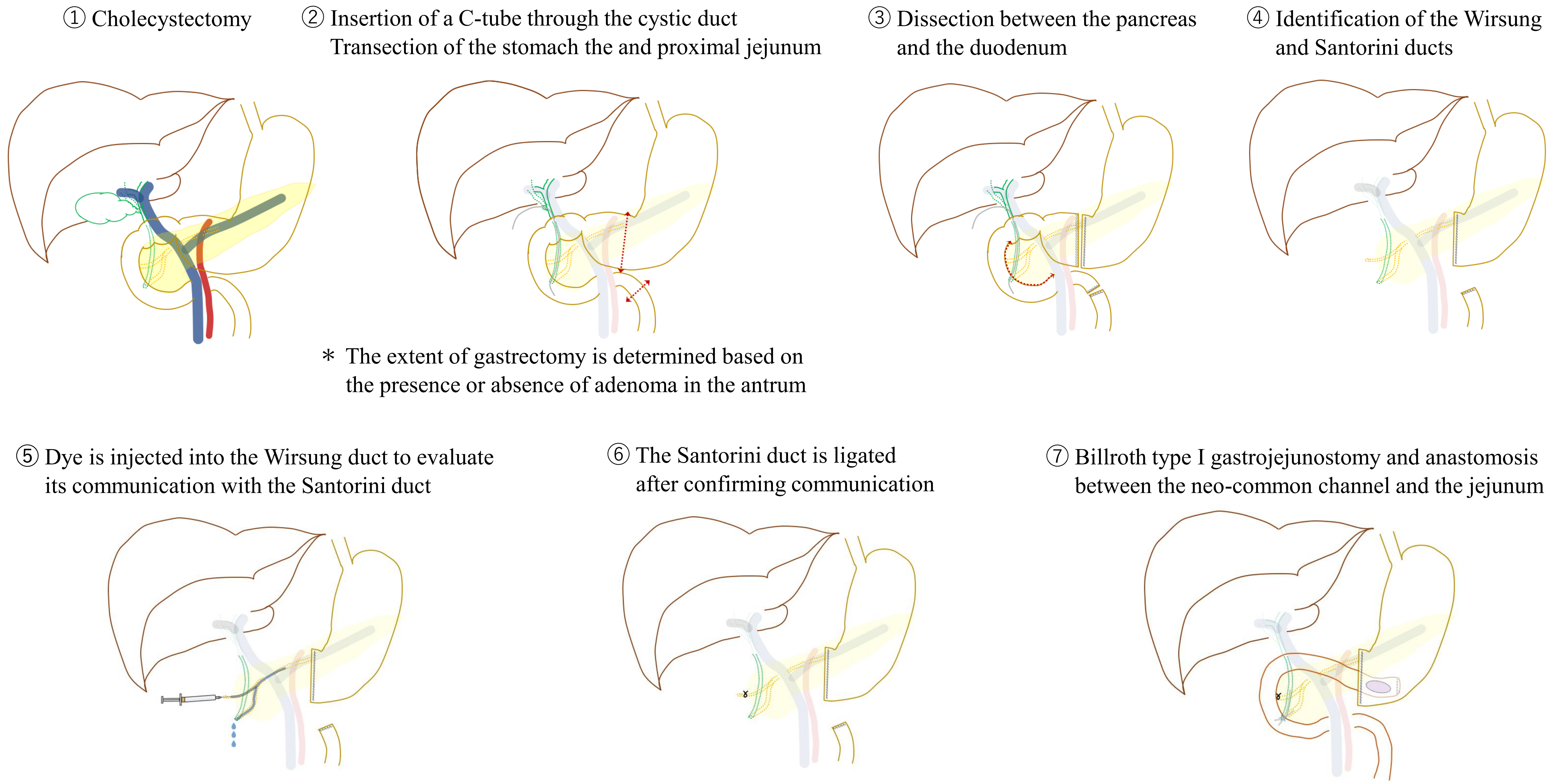

These figures were adapted and modified from
Ishida H, et al. Key points of daily care for familial adenomatous polyposis. Gastroenterological Surgery (Shoukakigeka). 2023;46(2):189-199. (in Japanese)
